# Identification of an Endoglin Variant Associated With HCV-Related Liver Fibrosis Progression by Next-Generation Sequencing

**DOI:** 10.3389/fgene.2019.01024

**Published:** 2019-11-04

**Authors:** Frédégonde About, Stéphanie Bibert, Emmanuelle Jouanguy, Bertrand Nalpas, Lazaro Lorenzo, Vimel Rattina, Mohammed Zarhrate, Sylvain Hanein, Mona Munteanu, Beat Müllhaupt, David Semela, Nasser Semmo, Jean-Laurent Casanova, Ioannis Theodorou, Philippe Sultanik, Thierry Poynard, Stanislas Pol, Pierre-Yves Bochud, Aurélie Cobat, Laurent Abel, The Swiss Hepatitis C Cohort Study Group, Francesco Negro, The French ANRS HC EP 26 Genoscan Study Group, Laurence Bousquet

**Affiliations:** ^1^Laboratory of Human Genetics of Infectious Diseases, Necker Branch, Inserm U1163, Paris, France; ^2^Paris Descartes University, Imagine Institute, Paris, France; ^3^Infectious Diseases Service, University Hospital and University of Lausanne, Lausanne, Switzerland; ^4^Inserm Scientific Information and Communication Department, Inserm, Paris, France; ^5^Genomics Core Facility, Imagine Institute, Research Federative Structure Necker, Inserm U1163 and Inserm US24/CNRS UMS3633, Paris Descartes Sorbonne Paris Cite University, Paris, France; ^6^Translational Genetics Platform, Inserm U1163, Imagine Institute, Paris Descartes University, Paris, France; ^7^BioPredictive Research, Paris, France; ^8^Gastroenterology and Hepatology Service, University Hospital of Zürich, Zürich, Switzerland; ^9^Division of Gastroenterology and Hepatology, Kantonsspital Sankt Gallen, Sankt Gallen, Switzerland; ^10^Department of Visceral Surgery and Medicine, Department of Hepatology, Inselspital Bern, Bern, Switzerland; ^11^St. Giles Laboratory of Human Genetics of Infectious Diseases, Rockefeller Branch, Rockefeller University, New York, NY, United States; ^12^Howard Hughes Medical Institute, New York, NY, United States; ^13^Pediatric Hematology-Immunology Unit, Necker Hospital for Sick Children, AP-HP, Paris, France; ^14^Center for Immunology and Infectious Diseases, Inserm UMR S 1135, Pierre et Marie Curie University, Paris, France; ^15^Université Paris Centre; U1223, Institut Pasteur; Liver Department, Hôpital Cochin, APHP; Paris, France; ^16^Hepatology Department, Assistance Publique-Hôpitaux de Paris, Pitié-Salpétrière Hospital, Paris, France; ^17^Saint-Antoine Research Center & Institute of Cardiometabolism and Nutrition (ICAN), Inserm, Sorbonne University, Paris, France

**Keywords:** endoglin, HCV-related liver fibrosis, exome sequencing, TGF-beta, rare-variant association study

## Abstract

Despite the astonishing progress in treating chronic hepatitis C virus (HCV) infection with direct-acting antiviral agents, liver fibrosis remains a major health concern in HCV infected patients, in particular due to the treatment cost and insufficient HCV screening in many countries. Only a fraction of patients with chronic HCV infection develop liver fibrosis. While there is evidence that host genetic factors are involved in the development of liver fibrosis, the common variants identified so far, in particular by genome-wide association studies, were found to have limited effects. Here, we conducted an exome association study in 88 highly selected HCV-infected patients with and without fibrosis. A strategy focusing on TGF-β pathway genes revealed an enrichment in rare variants of the endoglin gene (*ENG*) in fibrosis patients. Replication studies in additional cohorts (617 patients) identified one specific *ENG* variant, Thr5Met, with an overall odds ratio for fibrosis development in carriers of 3.04 (1.39–6.69). Our results suggest that endoglin, a key player in TGF-β signaling, is involved in HCV-related liver fibrogenesis.

## Introduction

End-stage chronic hepatitis C is the leading cause of liver transplantation in developed countries, despite the advent of potent antiviral drugs (Chhatwal et al., 2016). The pathogenesis of severe complications of chronic HCV infection remains unclear (Chhatwal et al., 2016). Most individuals with chronic HCV infection never develop cirrhosis, whereas others develop severe liver fibrosis in less than 20 years (Patin et al., 2012; Rueger et al., 2015). Known common risk factors for liver fibrosis progression include several host characteristics (e.g., male sex, older age at infection, HIV/HBV co-infection, metabolic syndrome, alcohol consumption) and viral factors (e.g., genotype 3) (Rueger et al., 2015). However, these factors explain only a small proportion of the inter-individual variability in liver fibrosis development (Patin et al., 2012), and there is evidence for the involvement of largely unidentified human genetic factors in fibrosis progression (Matsuura and Tanaka, 2018).

Several association studies have assessed the impact of common variants (polymorphisms) on liver fibrosis progression (Matsuura and Tanaka, 2018). In particular, two large genome-wide association studies (GWAS) on liver fibrosis have been conducted in HCV-infected HIV-negative (i.e., mono-infected) patients. The first, in a Caucasian population, identified four susceptibility loci, three of which are related to genes involved in apoptosis (Patin et al., 2012). The second, conducted in Japan, detected variants within the HLA region (Urabe et al., 2013). Finally, a GWAS in Caucasian patients with HCV/HIV co-infection detected another locus on chromosome 3p25 (Ulveling et al., 2016). These common variants have limited effects and could not explain the considerable variability in liver fibrosis development. Lower-frequency variants with stronger individual effects, particularly in coding regions, may instead be involved, and this hypothesis can now be tested by whole-exome sequencing (WES).

Transforming growth factor-β (TGF-β) is a key regulator of fibrosis (Meng et al., 2016) and is strongly involved in chronic liver disease contributing to all stages of disease progression from initial liver injury to fibrosis, cirrhosis, and hepato-cellular carcinoma (Dooley and ten Dijke, 2012; Fabregat et al., 2016). High levels of TGF-β result in activation of hepatic stellate cells (HSC) to myofibroblasts and massive hepatocyte cell death, which contributes to the promotion of liver fibrosis and later cirrhosis (Tsuchida and Friedman, 2017; Bansal and Chamroonkul, 2019). TGF-β1 promotes fibrosis through the well described TGF-β1/TGFBR1 canonical pathway (Fabregat et al., 2016; Meng et al., 2016). TGF-β1 binds to TGF-β receptor 2 (TGFBR2) which recruits and activates TGFBR1 (also known as ALK5). Active TGFBR1 phosphorylates Smad2 and Smad3 which form a complex with Smad4. These heteromeric complexes translocate to the nucleus to stimulate transcription of profibrotic molecules. Inhibitory Smad7 is a negative regulator of Smad2 and Smad3. In the present work, we focused on the TGF-β pathway, and we report here the results of a case-control exome-based association study investigating the role of rare and low frequency variants in 707 genes connected to this pathway in a cohort of highly selected patients with chronic HCV infection.

## Materials and Methods

### Study Subjects

Patients of the discovery cohort were selected from the French cohort of a previous GWAS of liver fibrosis progression in HCV mono-infected patients (Patin et al., 2012) [the ANRS Genoscan Study Cohort described elsewhere (Patin et al., 2012; Ulveling et al., 2016)]. We selected patients from Paris Hospitals (Cochin and La Pitié-Salpêtrière) with an extreme fibrosis phenotype determined by the examination of a liver biopsy specimen and the duration of HCV infection until liver biopsy. The severe fibrosis group included 48 patients (cases) who developed severe fibrosis (METAVIR score F3 or F4) in less than 30 years after the presumed date of HCV infection (mean duration = 18.8 y; SD = 6.8 y) without major comorbidities. The control group included 40 patients who did not develop fibrosis (METAVIR score F0 or F1) more than 20 years after the presumed date of HCV infection in the absence of treatment (mean = 30.3 y; SD = 7.8 y; **Table 1**).

### Whole Exome Sequencing (WES) and Variant Detection

WES was performed on an Illumina HiSeq 2500 by Agilent SureSelect All Exons V4 + UTRs (71 Mb) Single-Sample Capture at the Genomics Core Facility of the Imagine Institute (Paris, France). We used the Genome Analysis Software Kit (DePristo et al., 2011) (GATK v3.3) best practice pipeline to analyze our WES data. Reads were aligned with the human reference genome (hg19) with the maximum exact matches algorithm in Burrows–Wheeler Aligner (Li and Durbin, 2010). Local realignment around indels were performed with the GATK (McKenna et al., 2010). PCR duplicates were removed with Picard tools (broadinstitute.github.io/picard/). The GATK base quality score recalibrator (BQSR) was applied to correct sequencing artifacts. Individual genomic variant call files (gVCF) were generated with GATK HaplotypeCaller, and joint genotyping was performed with GATK GenotypeGVCFs. The calling process targeted regions covered by the WES Agilent 71-Mb Kit, including 200 bps flanking each region.

### Variant Level Quality Control

We filtered out sample genotypes with a coverage < 8X, a genotype quality (GQ) < 20, or a ratio of reads for the less covered allele (reference or variant allele) over the total number of reads covering the position (minor read ratio, MRR) < 20%, using an in-house script (Belkadi et al., 2015). We filtered out variant sites (i) multi-allelic (> 2 alleles), (ii) with more than 5% of missing genotypes, and (iii) flagged as probable false-positive variant sites by GATK Variant Quality Score Recalibrator (VQSR). The VQSR model was trained and applied for single nucleotide variants (SNV) and indels following the GATK best practices. We seek to achieve 99 and 95% sensitivities to the accessible sites in the truth set for SNVs and indels, respectively. A set of 439,790 high quality variants was retained for the analysis.

### Sample Level Quality Control

We required that each individual sample had a minimum 8X coverage for at least 80% of the targeted bases. We checked that reported sex was concordant with that inferred from sequence data. We further look at the distribution of the transition/transversion (Ti/Tv) ratio, the het/nonref-hom ratio, the indels/SNPs ratio, and the call rate for high quality variants in consensus coding regions. The median Ti/Tv ratio was 3.14, ranging from 3.07 to 3.24, as expected for coding regions. The median het/nonref-hom ratio was 1.59, ranging from 1.37 to 1.83, as expected for samples mainly from European origin (Wang et al., 2015). The median indel/SNP ratio was 0.017, ranging from 0.016 to 0.019 and consistent with the 1:43 ratio previously observed in coding regions (Ng et al., 2008). Finally, all the samples had a final call rate greater than 90% in coding regions and were kept for further analyses. Principal component analysis of our 88 samples with 1,000 genomes samples using high quality variants with minor allele frequency in the cohort ≥ 0.02 (Belkadi et al., 2016) confirmed that most patients were of European origin except five patients. Four patients (two cases and two controls) were of North African origin, and one case was very likely of mixed European and Asian origin (**Supplementary Figure 1**). We decided to keep those five patients and to adjust all association analyses on the three first principal components.

### Variant Selection

We focus our analyses on the most likely functionally relevant variants based on several variant annotations. Variant effects were predicted using snpEff v4.2 (Cingolani et al., 2012) and the Ensembl GRCh37.75 reference database. For variants that have different annotations due to multiple transcripts of the gene, the highest impact effect for each variant was taken. Additional annotations including variant frequencies in ExAC populations (Lek et al., 2016), the Combined Annotation Dependent Depletion (CADD) score (Kircher et al., 2014), and the gene specific Mutation Significance Cutoff (MSC) (Itan et al., 2016) were performed using variant tools (San Lucas et al., 2012). Candidate coding variants (CCV) were finally defined as missense, splice acceptor, splice donor, stop gained, stop loss, start loss, initiator codon variant, exon loss, frameshift, and in-frame insertions/deletions with a CADD score greater than the MSC, and with an ExAC frequency in the European non-Finnish population below 0.05.

### Gene Selection

In the present work, we focused on the TGF-β signaling pathway, the central driver of liver fibrosis (Meng et al., 2016). We selected the seven main core genes of this pathway: *TGFB1*, *TGFBR1*, *TGFBR2*, *SMAD2*, *SMAD3*, *SMAD4*, and *SMAD7* and further increased the list of genes of interest to 707 autosomal genes directly connected to those 7 core genes according to the human gene connectome (Itan et al., 2013), i.e., we retained only the genes connected to at least one of the 7 main core genes at a degree of connectivity of 1 (**Supplementary Table 1**). Within those 707 genes of interest, we identified 1,243 high quality CCV as defined in the variant selection section.

### Statistical Analysis

To test whether rare candidate coding variants contribute to fibrosis, we collapsed candidate coding variants into a single burden variable which takes the value 1 if the patient carries at least one CCV and 0 otherwise and we compared the aggregated carrier frequency of candidate coding variants between cases and controls by logistic regression, as implemented in the R software using the glm function. Since principal component analysis identified five patients of non-European origin (**Supplementary Figure 1**), the analyses were adjusted on the three first principal components. We overcame the problem of the small sample size by restricting our analysis to 116 genes for which candidate coding variants were carried by more than five individuals leading to a Bonferroni corrected threshold of p < 0.00043 to account for multiple testing.

### Replication Studies

To replicate our results, we first performed targeted next-generation sequencing focusing on the coding regions of the endoglin gene on the same Illumina HiSeq2500 using a customized SureSelect gene panel in 161 additional Caucasian HCV mono-infected patients from the ANRS Genoscan Study Cohort, including 59 who developed severe liver fibrosis (METAVIR F3–F4) after a mean duration of 16.5 years of infection (SD = 5.5 years), and 102 patients who were fibrosis-free (METAVIR F0-F1) after a mean duration of infection of 25.9 y (SD = 5.8 years; **Table 1**).

We further investigated the association of rs35400405 with fibrosis in HCV mono-infected patients, in an additional cohort of 456 patients (308 severe fibrosis patients with METAVIR F3–F4 and 148 fibrosis-free patients with METAVIR F0–F1) of Caucasian origin from Switzerland (the Swiss Hepatitis C Cohort Study) with chronic HCV mono-infection (**Table 1**). In this cohort, rs35400405 was genotyped using a fluorescent based competitive allele specific polymerase chain reaction (KASP, LGC Genomics, Hoddesdon, Herts, United Kingdom). All the methods used were performed in accordance with relevant guidelines and regulations.

## Results

### Discovery Cohort

**Figure 1:**
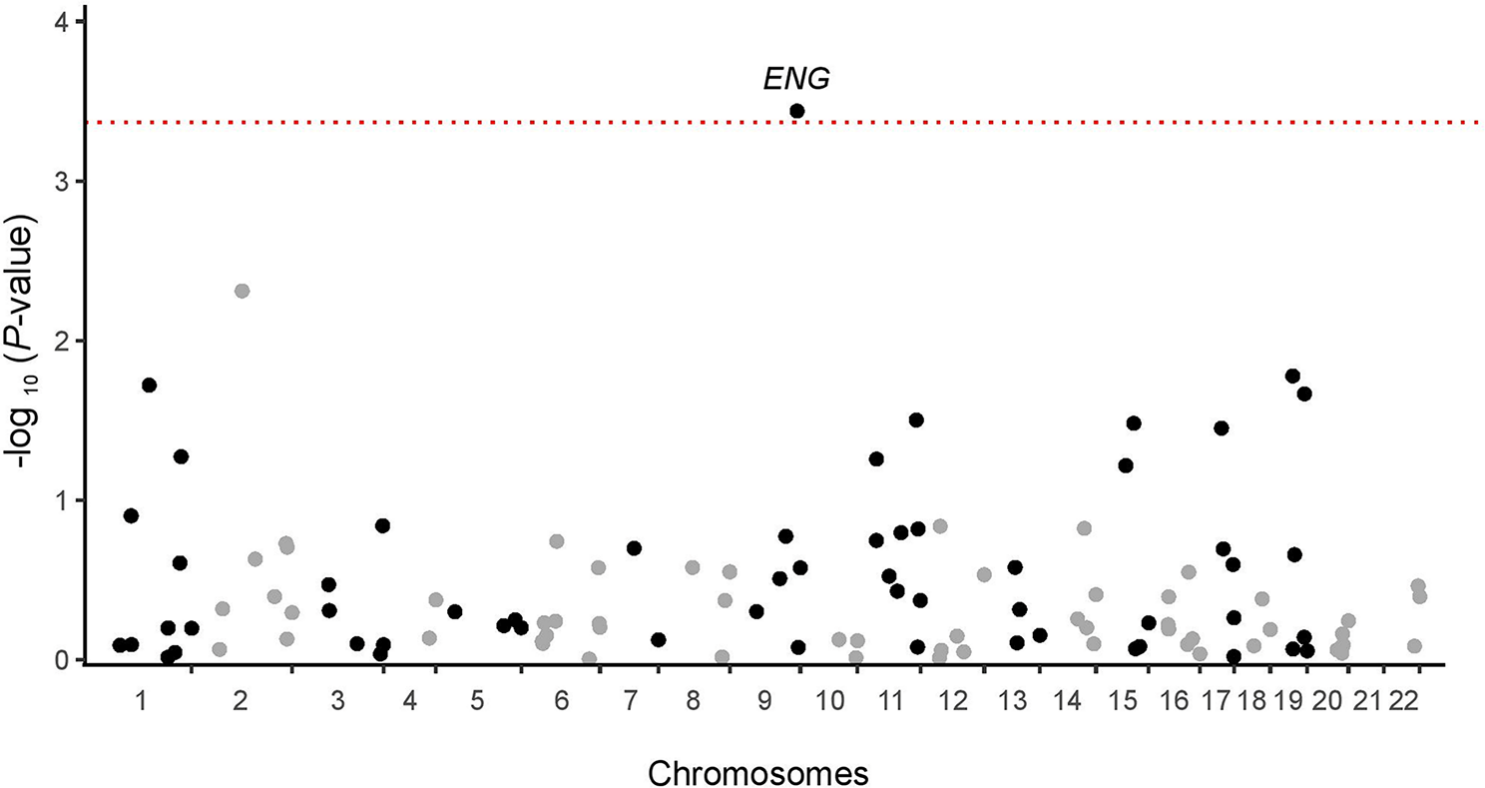
Manhattan plot for the exome association analysis of 116 informative TGF-β pathway genes in the discovery cohort. The dotted red line represents the significance threshold after Bonferroni correction (p = 0.00043).

Results of the exome association study in the discovery cohort of 88 patients for the 116 genes of the TGF-β pathway with more than 5 CCV carriers are shown in **Figure 1**. The main signal observed was that for *ENG*, encoding endoglin, with a *P*-value of 0.00037, which remained significant after Bonferroni correction (*P*-value_corrected_ = 0.043). The *P*-value was an order of magnitude larger for the next most closely associated gene (**Supplementary Table 2**). For *ENG*, there were seven heterozygous carriers of at least one CCV in the severe fibrosis group and none in the control group. There were only three *ENG* candidate coding variants: Thr5Met found in four patients, Gly191Asp in two patients, and Pro131Leu in one patient (**Supplementary Table 3**). Six of the seven carriers were of European origin, and one, carrying Gly191Asp, was of mixed ancestry, likely Eurasian (**Supplementary Figure 1**).

#### Replication Studies

For replication of these results, we performed targeted next-generation sequencing focusing on the coding regions of *ENG* in 161 additional Caucasian HCV mono-infected patients. No significant enrichment in *ENG* candidate coding variants was found in this replication cohort, mostly due to the presence of three novel rare candidate coding variants in the control group (**Supplementary Table 3**). However, the frequency of carriers of the most common CCV enriched in our discovery cohort, Thr5Met (c.14C > T), was higher among fibrosis patients (10.2%) than among controls (3.9%) in the replication cohort although not significant (*P*-value = 0.12).

We conducted a second replication study by genotyping Thr5Met (rs35400405) in a larger independent Swiss cohort of HCV mono-infected patients including 308 patients with liver fibrosis and 148 without fibrosis (**Table 1**). Thr5Met was found to be significantly more abundant (*P*-value = 0.032) in fibrosis patients (7.5%) than in controls (2.7%). Overall, when all cohorts were combined, the frequency of Thr5Met carriers was 8.0% in fibrosis patients and 2.8% in controls, resulting in an odds ratio of 3.04 (1.39–6.69; *P*-value = 0.0024; **Table 2**). The frequency of Thr5Met carriers in >50,000 Europeans in the gnomAD database (http://gnomad.broadinstitute.org/) was 4.1%, confirming an enrichment of this variant in fibrosis patients, and a depletion in those without fibrosis.

**Table 1:** Clinical and demographic characteristics of chronically HCV infected patients of the discovery cohort (N = 88), the French replication cohort (N = 161), and the Swiss replication cohort (N = 453).

Covariate	Category	Discovery cohort			French replication cohort			Swiss replication cohort		
		No fibrosis	Severe fibrosis	Total (%)	No fibrosis	Severe fibrosis	Total (%)	No fibrosis	Severe fibrosis	Total (%)
		N = 40	N = 48	N = 88	N = 102	N = 59	N = 161	N = 148	N = 308	N = 456
Sex	Female	22	25	47 (53.4%)	61	22	83 (51.5%)	74	90	164 (36%)
METAVIR score	F0	13	–	13 (14.8%)	15	–	15 (9.3%)	39	–	39 (9%)
	F1	27	–	27 (30.7%)	87	–	87 (54.0%)	106	–	106 (23%)
	F3	–	32	32 (36.3%)	–	30	30 (18.6%)	–	128	128 (28%)
	F4	–	16	16 (18.2%)	–	29	29 (18.0%)	–	180	180 (40%)
Alcohol consumption (^a^)	Low	40	42	82 (93.1%)	91	40	131 (81.4%)	65	126	191 (42%)
	High	–	7	7 (7.9%)	11	19	30 (18.6%)	80	181	261 (58%)
HCV mode of acquisition	IDU	9	16	25 (28.4%)	39	21	60 (37.3%)	54	101	155 (34%)
	Blood transfusion	22	23	45 (51.1%)	41	29	70 (43.4%)	35	60	95 (21%)
	Other/NA	9	9	18 (20.5%)	22	9	31 (19.3%)	56	147	203 (45%)
HCV genotype	1	25	34	59 (67.0%)	77	39	116 (72.0%)	90	142	232 (51%)
	2	9	1	10 (11.4%)	6	2	8 (5.0%)	13	38	51 (11%)
	3	1	8	9 (10.2%)	13	12	25 (15.5%)	24	91	115 (25%)
	4	1	2	3 (3.4%)	–	–	–	16	30	46 (10%)
	Other/NA	4	3	7 (7.9%)	6	6	12 (7.5%)	2	7	9 (2%)
Age at infection (y) (^b^)	Median (Q1–Q3)	18 (11.5–23)	29 (22–39)		21 (17–27)	36 (21–46)		15 (8–18.5)	23 (17–27.5)	
Duration of infection (y) (^c^)	Median (Q1–Q3)	30 (24–34.5)	18 (14–26)		24 (21–29)	16 (12–19)		32 (27–36)	25 (18–31)	

(^a^)The threshold to define high alcohol consumption in the French cohorts and in the Swiss replication cohort were ≥30g/d and ≥40g/d, respectively. Alcohol consumption was missing in one patient with severe liver fibrosis in the Swiss replication cohort.

(^b^)Age at infection was missing in 77 patients with severe liver fibrosis in the Swiss replication cohort.

(^c^)Duration of infection was missing in 77 patients with severe liver fibrosis in the Swiss replication cohort.

**Table 2:** Distribution and overall effect of *ENG* Thr5Met on liver fibrosis progression in the discovery cohort and the French and Swiss replication cohorts.

	Discovery		French replication		Swiss replication		All Cohorts		OR [95%CI] ^(a)^	*P*-value^(a)^
	No fibrosis	Severe fibrosis	No fibrosis	Severe fibrosis	No fibrosis	Severe fibrosis	No fibrosis	Severe fibrosis		
	N = 40	N = 48	N = 102	N = 59	N = 148	N = 308	N = 290	N = 415		
N carriers^(b)^ (%)	0 (0)	4 (8.3%)	4 (3.9%)	6 (10.2%)	4 (2.7%)	23 (7.5%)	8 (2.8%)	33 (8.0%)	3.04 (1.39–6.69)	0.0024

^(a)^Computed by means of the likelihood ratio test in the logistic regression framework.

^(b)^All carriers were heterozygous for Thr5Met.

#### ***In Silico*** Analysis of Thr5Met Variant

**Figure 2:**
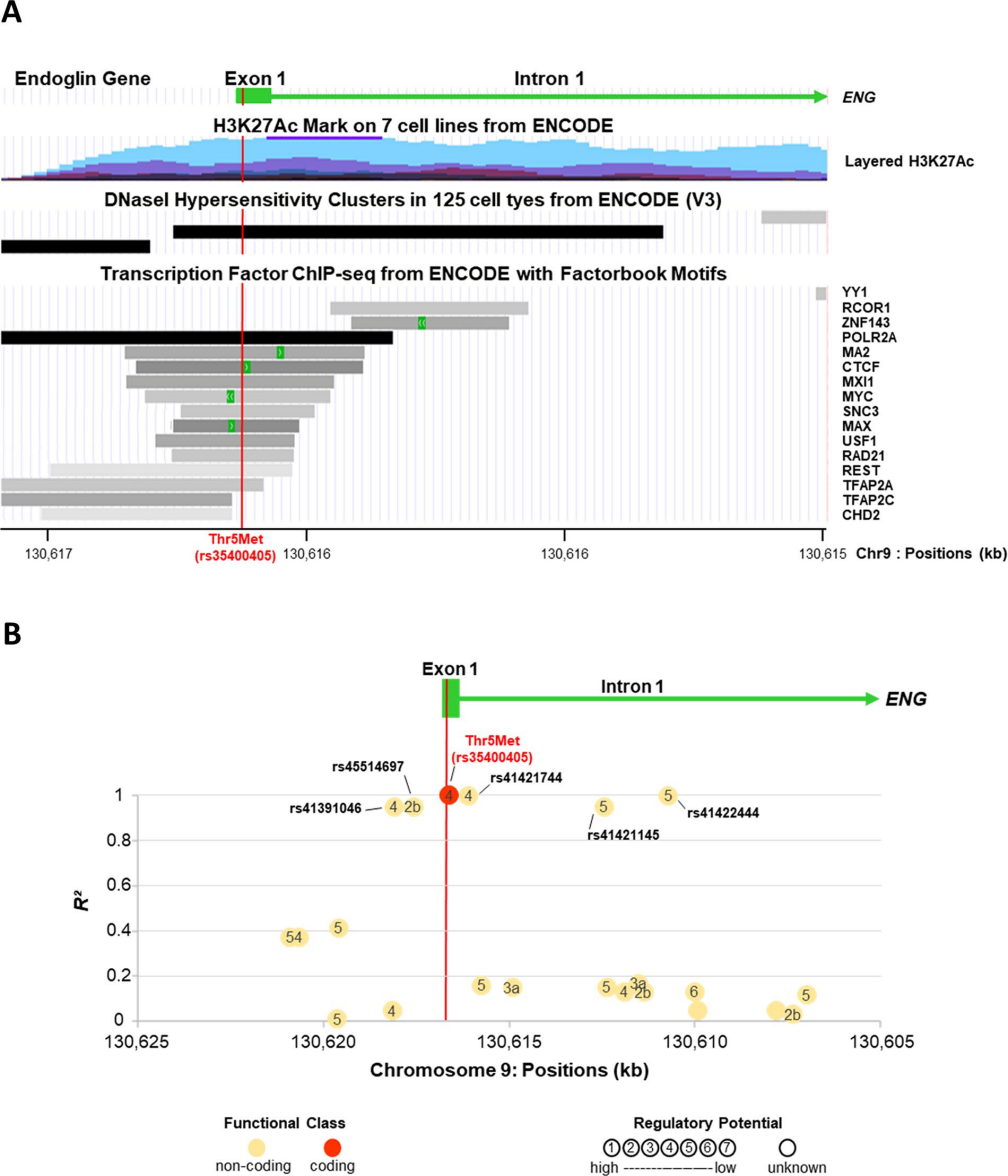
*ENG* variant Thr5Met (rs35400405) is in a regulatory region and in strong linkage disequilibrium with a potential regulatory SNP, rs45514697. The X axis for chromosome 9 positions is presented in the same direction as the transcription of *ENG*. **(A)** Eng Variant Thr5met (Rs35400405) Is in a Regulatory Region. Figure is modified from UCSC Genome Browser on Human hg19/GRCh37 Assembly (http://genome.cse.ucsc.edu). The H3K27Ac histone mark is the acetylation of lysine 27 of the H3 histone protein. It is reported as an important enhancer mark that can distinguish between active and poised enhancer elements (Creyghton et al., 2010). Schema for layered H3K27Ac shows that ENG variant Thr5Met is in an active regulatory region. Schema of DNaseI hypersensitivity clusters confirms that ENG variant Thr5Met is in a region with high transcriptional activity. Moreover, several transcription factors are found to bind to this same region. **(B)** Linkage Disequilibrium (Ld) Patterns of Snps Located in the Vicinity of Thr5met (Rs35400405). The Y axis shows the LD as measured by R². SNPs are indicated by circles and numbers within circles indicate the potential regulatory function of the SNP as predicted by RegulomeDB score (http://www.regulomedb.org/). ENG variant Thr5Met (rs35400405) is in almost perfect linkage disequilibrium with five non-coding SNPs (R² from 0.95 to 1). One of them, rs45514697 (R² = 0.95), is likely to affect binding (RegulomeDB score = 2b).

The Thr5Met variant maps to exon 1 of *ENG* encoding the signal peptide (**Figure 2A**). Thr5Met was predicted to be benign by PolyPhen and tolerated by SIFT, but it is in very strong linkage disequilibrium with five SNPs (**Figure 2B**) one of which, rs45514697, is predicted to affect transcription factor binding (www.regulomedb.org). SNP rs45514697 is predicted to modify a regulatory motif for liver factor (LF)-A1 (also known as hepatocyte nuclear factor 4), which is involved in the expression of several liver-specific genes (Ramji et al., 1991), with a lower affinity for the rare allele (http://archive.broadinstitute.org/mammals/haploreg/haploreg.php).

## Discussion

In the present study, we used a three-stage analysis to identify potential protein-coding variants that contribute to liver fibrosis development in HCV mono-infected individuals. In contrast to GWAS investigating common variants, we focused on more rare potentially damaging coding variants identified by WES in 707 candidate genes linked to the TGF- β signaling pathway. We identified one low frequency missense variant (Thr5Met) in *ENG* gene, encoding endoglin, associated with liver fibrosis development. Very interestingly, this variant is also in perfect linkage disequilibrium with rs45514697, an upstream *ENG* variant located in a regulatory region. The rare allele of rs45514697 which is at risk for liver fibrosis is predicted to strongly decrease the DNA-binding affinity for LF-A1, a transcription factor involved in the expression of several liver specific genes (Ramji et al., 1991).

Endoglin, also known as cluster of differentiation CD105, is a TGF-β co-receptor. Heterozygous LOF mutations of *ENG* cause hereditary hemorrhagic telangiectasia type 1 (HHT1), also known as Osler–Weber–Rendu disease, a rare autosomal dominant condition characterized by telangiectasias and arteriovenous malformations (Meurer et al., 2014). Interestingly, the three variants identified in the discovery cohort, Thr5Met, Pro131Leu, and Gly191Asp, were previously observed in HHT1 patients (Cymerman et al., 2003; Abdalla et al., 2005; Fernandez et al., 2006; Olivieri et al., 2007; Fontalba et al., 2008). Variants Thr5Met and Gly191Asp are presently considered as non-pathogenic polymorphisms (Abdalla and Letarte, 2006; Olivieri et al., 2007), consistently with their frequency in public databases (MAF > 1% in gnomAD European population). The pathogenic role of Pro131Leu in HHT is more debated as the variant is more rare (MAF = 0.001 in the gnomAD European population), was associated with reduced levels of endoglin (Cymerman et al., 2003; Fernandez et al., 2006), but was also found in healthy individuals (Abdalla and Letarte, 2006; Olivieri et al., 2007). We did not have direct information about HHT symptoms (such as epistaxis or telangiectasia) for the patients carrying *ENG* variants in our cohort. However, they had no contraindication to liver biopsy, and no signs of arterio-venous malformations were reported in the biopsy. Of note, 41–74% of HHT patients present liver involvement with hepatic vascular malformations (Buscarini et al., 2018). Livers with HHT can show nodular hyperplasia and fibrosis around the abnormal vessels which, associated with portal hypertension, may lead to a misdiagnosis of cirrhosis (Buscarini et al., 2018).

In addition to its well-established role in angiogenesis, there is evidence to suggest that endoglin is involved in fibrogenesis (Meurer et al., 2014). Endoglin is strongly expressed in profibrogenic cell types such as mesangial cells, cardiac fibroblasts, scleroderma fibroblasts, and HSCs which are the most fibrogenic cells of the liver. Indeed, endoglin plays a crucial role in TGF-β signaling and is thought to modulate the balance between the main profibrotic TGF-β1/TFBR1 signaling pathway and the TGF-β1/ACVRL1 signaling pathway (Velasco et al., 2008), which has a less studied and more controversial role in fibrosis (Munoz-Felix et al., 2013). There are two isoforms of endoglin resulting from alternative splicing, the long (L-Eng) and the short (S-Eng) endoglin (Gougos and Letarte, 1990; Bellon et al., 1993). It was previously found that L-Eng, the predominant form, enhances ACVRL1 (also known as ALK1) (Velasco et al., 2008) while S-Eng promotes TGFBR1 (Blanco et al., 2008; Velasco et al., 2008) signaling pathways. It was also shown that endoglin mediates the crosstalk between the TGF-β and the fibronectin/integrin signaling pathways in endothelial cells (Tian et al., 2012). Interestingly, HCV core protein was recently reported to modulate endoglin signaling in liver pathogenesis by upregulating endoglin expression on cell surface and activating its downstream ACVRL1 signaling pathway (Kwon et al., 2017). In addition, patients with chronic HCV infection and liver cirrhosis were found to have high intrahepatic and circulating endoglin levels (Clemente et al., 2006).

Whether endoglin stimulates or inhibits fibrosis is still debated. Several *in vitro* studies have been conducted that support either hypothesis. In numerous cells types, such as mesangial cells, myoblasts, fibroblasts, or chondrocytes, endoglin regulates negatively TGF-β-induced extracellular matrix (ECM) protein expression (Diez-Marques et al., 2002; Guo et al., 2004; Obreo et al., 2004; Rodriguez-Barbero et al., 2006; Finnson and Philip, 2012). Other experiments outlined a profibrogenic role of endoglin in HSC cell lines (Meurer et al., 2011; Meurer et al., 2013) or in scleroderma patient fibroblasts (Morris et al., 2011). In an *in vivo* mouse model of kidney fibrosis following ureteral obstruction, L-Eng overexpression was associated with higher amounts of type I collagen and fibronectin (Oujo et al., 2014). More recently, endoglin haploinsufficiency in mice was shown to differentially regulate ECM production in skin and cartilage (Alzahrani et al., 2018). The conflicting results observed on the role of endoglin in fibrosis may lie in the respective contribution of the two splice variants L- and S-endoglin which differ in their cytoplasmic domain and could have antagonizing effects (Velasco et al., 2008; Munoz-Felix et al., 2016), and/or in differential effects according to tissues (Alzahrani et al., 2018) or experimental conditions.

In a more relevant murine model of liver fibrosis, endoglin deficiency in HSC was recently shown to significantly aggravate liver fibrosis in response to injury suggesting a protective role of endoglin against liver fibrosis (Alsamman et al., 2018). This is consistent with a putative hypomorphic role of the rare allele of rs45514697 in liver fibrosis risk, with a predicted decrease in affinity at the corresponding LF-A1 motif. Further functional studies of Thr5Met and rs45514697, in particular, are required in HSCs, to determine their role in endoglin expression and/or function and provide insight into the potential antifibrogenic role of endoglin in the liver. These results may open the way for new treatments aimed at targeting specific signaling pathways involved in liver fibrogenesis (Bansal and Chamroonkul, 2019).

## Data Availability Statement

The datasets generated for this study can be found in the dbGaP, accession phs001902.v1.p1, http://www.ncbi.nlm.nih.gov/projects/gap/cgi-bin/study.cgi?study_id=phs001902.v1.p1

## Ethics Statement

The studies involving human participants were reviewed and approved by the french Committee for the Protection of Persons (CPP) Île de France 3 of the Tarnier-Cochin Hospital (Paris, France) for the Genoscan study. The ethics committees of all participating centers granted ethical approval for the Swiss Hepatitis C Cohort Study: Ethics Committee, Medical Department, Zurich; Ethics Committee, Medical Department, Geneva; Ethics Committee for clinical research, Faculty of Biology and Medicine, Lausanne; Ethics Committee Canton St Gallen; Ethics Committee Canton of Bern; Ethics Committee Canton Ticino; Intercantonal Ethics Committee for Jura, Fribourg and Neuchatel; Ethics Committee of both Basel). The patients/participants provided their written informed consent to participate in this study.

## Author Contributions

AC and LA conceived and designed the study. BN, MM, BM, DS, NS, PS, TP, SP, and P-YB contributed to the recruitment of participants and the clinical measurements. MZ and SH carried out the sequencing of individuals. LL, EJ, VR, and IT contributed reagents/materials/analysis tools. FA and SB performed the data analysis. FA, AC, and LA interpreted the results and wrote the first draft of the manuscript. J-LC contributed to the manuscript in its final form. All authors reviewed the manuscript.

## Funding

The French cohort is supported and sponsored by The National Agency for Research on AIDS and Viral Hepatitis (ANRS) (ANRS Study HC EP 26 Genoscan). The SCCS is supported by grants from the Swiss National Science Foundation (3347C0-108782/1), the Swiss Federal Office for Education and Sciences (03.0599), and the European Commission (LSHM-CT-2004-503359; VIRGIL Network of Excellence on Antiviral Drug Resistance). P-YB was supported by the Swiss National Foundation (grant number 324730-144054); the Santos-Suarez Foundation and the Leenaards foundation. FA is the recipient of a fellowship from Fondation pour la Recherche Médicale (FRM, Grant n° FDM20140630671) The laboratory of Human Genetics of infectious Diseases is supported by The National Agency for Research on AIDS and Viral Hepatitis (ANRS), Institut National de la Santé et de la Recherche Médicale (INSERM), Paris Descartes University, the French National Research Agency (ANR) under the ‟Investments for the future” program (grant no. ANR-10-IAHU-01).

## Conflict of Interest

Author MM was employed by company BioPredictive Research, France.

The remaining authors declare that the research was conducted in the absence of any commercial or financial relationships that could be construed as a potential conflict of interest.
