# Supplementary material for: Identification of an Endoglin Variant Associated With HCV-Related Liver Fibrosis Progression by Next-Generation Sequencing
Source: Front Genet. 2019 Nov 4;10:1024. doi: 10.3389/fgene.2019.01024 (PMC6844190; doi:10.3389/fgene.2019.01024)
Supplement: Supplementary file 1 [file DataSheet_1.pdf]

Supplementary Material

1 Supplementary Figures

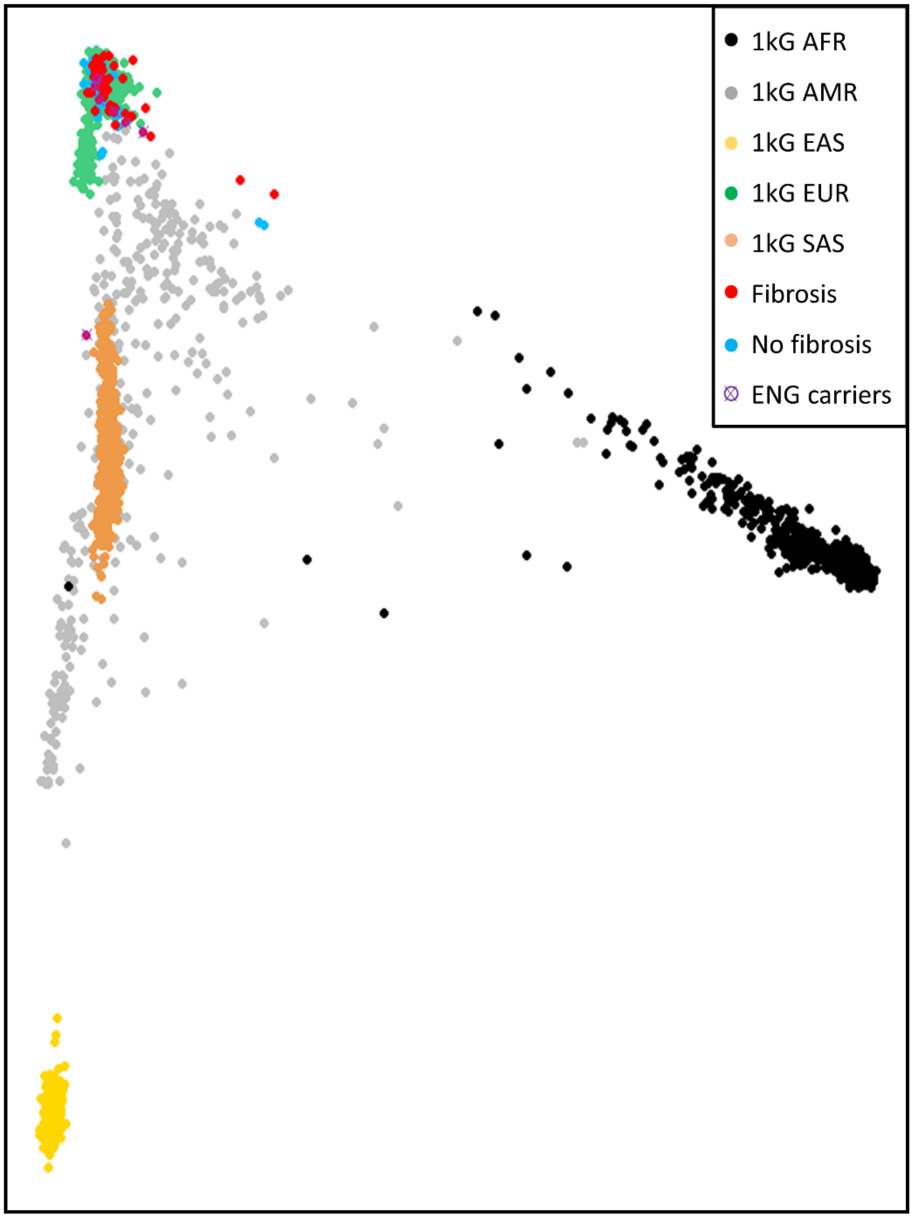

Supplementary Figure 1. Assessment of population structure in the discovery cohort by Principal Component Analysis (PCA) using 1000 Genomes (1kG) Project phase 3 populations

PCA was performed with Plink v1.9 software on Whole-Exome Sequencing data and 1000 Genomes (1kG) Project phase 3 public database as reference, selecting good quality variants (after filtering) with a minor allele frequency > 0.02 and a call rate > 0.95 (Belkadi et al., 2016). 1000 Genomes populations were classified into 4 groups: African (AFR, i.e. YRI, LWK, ASW), American (AMR, i.e. MXL, CLM, and PUR), East Asian (CHB, JPT and CHS) and European (CEU, TSI, GBR, FIN, IBS) populations. Our sample of patients with severe fibrosis (in red) and without fibrosis (in light blue) is mainly of European ancestry, except 5 patients. Four were of North African origin (2 cases and 2 controls) and one was very likely of mixed European and Asian origin.

2 Supplementary Tables

Supplementary table 1. List of the 707 autosomal genes connected to the 7 main core genes of the TGF-β pathway at a degree of connectivity of 1.

Target genes	Chr	Gene name	Main core genes of TGF-β pathway ([a])							Total of core genes ([b])
N=707			TGFB1	TGFBR1	TGFBR2	SMAD2	SMAD3	SMAD4	SMAD7	
A2M	12	alpha-2-macroglobulin	X							1
ACTA1	1	actin, alpha 1, skeletal muscle	X							1
ACTA2	10	actin, alpha 2, smooth muscle, aorta	X							1
ACTB	7	actin beta					X			1
ACTN1	14	actinin alpha 1	X							1
ACTN2	1	actinin alpha 2	X							1
ACTN4	19	actinin alpha 4	X							1
ACTR1B	2	ARP1 actin related protein 1 homolog B				X				1
ACVR1	2	activin A receptor type 1		X						1
ACVR1C	2	activin A receptor type 1C		X				X		2
ACVR2A	2	activin A receptor type 2A							X	1
ACVR2B	3	activin A receptor type 2B				X			X	2
ACVRL1	12	activin A receptor like type 1	X	X						2
ADAMTS3	4	ADAM metallopeptidase with thrombospondin type 1 motif 3	X							1
ADAMTS4	1	ADAM metallopeptidase with thrombospondin type 1 motif 4	X							1
ADH1A	4	alcohol dehydrogenase 1A (class I), alpha polypeptide							X	1
AGL	1	amylo-alpha-1, 6-glucosidase, 4-alpha-glucanotransferase					X			1
AKT1	14	AKT serine/threonine kinase 1	X	X		X	X	X	X	6
ALB	4	albumin	X						X	2
ALDOA	16	aldolase, fructose-bisphosphate A	X							1
ALYREF	17	Aly/REF export factor						X		1

ANAPC10	4	anaphase promoting complex subunit 10				X	X			2
ANGPT1	8	angiopoietin 1	X							1
ANK1	8	ankyrin 1		X						1
ANXA2	15	annexin A2	X							1
AP2B1	17	adaptor related protein complex 2 beta 1 subunit		X	X					2
APOE	19	apolipoprotein E	X							1
APP	21	amyloid beta precursor protein	X							1
ARHGEF18	19	Rho/Rac guanine nucleotide exchange factor 18	X	X	X					3
ARID1A	1	AT-rich interaction domain 1A				X	X			2
ARID1B	6	AT-rich interaction domain 1B				X				1
ARNT	1	aryl hydrocarbon receptor nuclear translocator					X	X		2
ASCL1	12	achaete-scute family bHLH transcription factor 1						X		1
ASPN	9	asporin					X			1
ATAD3B	1	ATPase family, AAA domain containing 3B				X				1
ATF2	2	activating transcription factor 2					X	X		2
ATF3	1	activating transcription factor 3				X	X	X		3
ATM	11	ATM serine/threonine kinase							X	1
AXIN1	16	axin 1		X	X		X		X	4
AXIN2	17	axin 2							X	1
BACH1	21	BTB domain and CNC homolog 1					X			1
BAMBI	10	BMP and activin membrane bound inhibitor	X	X	X				X	4
BCAR1	16	BCAR1, Cas family scaffolding protein					X			1
BCL2	18	BCL2, apoptosis regulator	X							1
BCL6	3	B cell CLL/lymphoma 6					X	X		2

BGLAP	1	bone gamma-carboxyglutamate protein	X			X				2
BIRC5	17	baculoviral IAP repeat containing 5	X							1
BLZF1	1	basic leucine zipper nuclear factor 1					X			1
BMP2	20	bone morphogenetic protein 2	X					X	X	3
BMP4	14	bone morphogenetic protein 4						X	X	2
BMP5	6	bone morphogenetic protein 5		X						1
BMP6	6	bone morphogenetic protein 6							X	1
BMP7	20	bone morphogenetic protein 7							X	1
BMPR1B	4	bone morphogenetic protein receptor type 1B							X	1
BMPR2	2	bone morphogenetic protein receptor type 2				X			X	2
BRCA1	17	BRCA1, DNA repair associated					X			1
BRCA2	13	BRCA2, DNA repair associated					X			1
BTRC	10	beta-transducin repeat containing E3 ubiquitin protein ligase					X	X		2
BUB1	2	BUB1 mitotic checkpoint serine/threonine kinase				X				1
C10orf118	10	coiled-coil domain containing 186					X			1
C1orf116	1	chromosome 1 open reading frame 116				X	X			2
C6orf132	6	chromosome 6 open reading frame 132						X		1
CAMK2A	5	calcium/calmodulin dependent protein kinase II alpha				X				1
CAV1	7	caveolin 1	X	X	X	X	X		X	6
CBFB	16	core-binding factor beta subunit					X	X		2
CBL	11	Cbl proto-oncogene			X		X			2
CCL17	16	C-C motif chemokine ligand 17							X	1
CCL2	17	C-C motif chemokine ligand 2	X							1
CCNB1	5	cyclin B1			X					1

CCNB2	15	cyclin B2			X					1
CCND1	11	cyclin D1			X			X		2
CCNG2	4	cyclin G2				X	X	X		3
CCNH	5	cyclin H		X	X					2
CCNT1	12	cyclin T1		X	X					2
CD209	19	CD209 molecule	X							1
CD226	18	CD226 molecule	X							1
CD44	11	CD44 molecule (Indian blood group)		X						1
CDC16	13	cell division cycle 16				X	X			2
CDC25A	3	cell division cycle 25A					X	X		2
CDC27	17	cell division cycle 27				X	X			2
CDC7	1	cell division cycle 7					X			1
CDH1	16	cadherin 1	X			X	X	X	X	5
CDH2	18	cadherin 2	X						X	2
CDK2	12	cyclin dependent kinase 2				X	X	X		3
CDK4	12	cyclin dependent kinase 4					X	X		2
CDK7	5	cyclin dependent kinase 7		X	X					2
CDK8	13	cyclin dependent kinase 8					X			1
CDK9	9	cyclin dependent kinase 9		X	X					2
CDKN1A	6	cyclin dependent kinase inhibitor 1A	X			X	X	X	X	5
CDKN1B	12	cyclin dependent kinase inhibitor 1B	X				X	X		3
CDKN2A	9	cyclin dependent kinase inhibitor 2A					X	X		2
CDKN2B	9	cyclin dependent kinase inhibitor 2B	X			X	X	X		4
CDX1	5	caudal type homeobox 1					X			1
CEBPA	19	CCAAT/enhancer binding protein alpha	X				X	X		3

CEBPB	20	CCAAT/enhancer binding protein beta				X	X	X		3
CEBPD	8	CCAAT/enhancer binding protein delta					X	X		2
CELF1	11	CUGBP Elav-like family member 1							X	1
CFD	19	complement factor D	X							1
CGN	1	cingulin	X	X	X					3
CHUK	10	conserved helix-loop-helix ubiquitous kinase		X	X	X	X			4
CIITA	16	class II major histocompatibility complex transactivator					X			1
CITED2	6	Cbp/p300 interacting transactivator with Glu/Asp rich carboxy-terminal domain 2				X	X			2
CLDN7	17	claudin 7						X		1
CLU	8	clusterin	X	X	X					3
COL1A2	7	collagen type I alpha 2 chain					X	X		2
COL2A1	12	collagen type II alpha 1 chain	X							1
COPS5	8	COP9 signalosome subunit 5						X		1
CREB1	2	cAMP responsive element binding protein 1	X				X			2
CREBBP	16	CREB binding protein				X	X	X		3
CRK	17	CRK proto-oncogene, adaptor protein					X			1
CSF2	5	colony stimulating factor 2							X	1
CSNK1D	17	casein kinase 1 delta				X	X	X		3
CSNK1G2	19	casein kinase 1 gamma 2					X			1
CSRP2	12	cysteine and glycine rich protein 2	X							1
CTBP1	4	C-terminal binding protein 1				X		X		2
CTCF	16	CCCTC-binding factor					X	X		2
CTDSP1	2	CTD small phosphatase 1				X	X			2
CTDSP2	12	CTD small phosphatase 2				X	X			2

CTDSPL	3	CTD small phosphatase like				X	X			2
CTGF	6	connective tissue growth factor	X	X	X	X	X	X	X	7
CTNNB1	3	catenin beta 1		X	X	X	X	X	X	6
CUL1	7	cullin 1				X	X			2
CXCL12	10	C-X-C motif chemokine ligand 12					X			1
CYLD	16	CYLD lysine 63 deubiquitinase							X	1
CYP17A1	10	cytochrome P450 family 17 subfamily A member 1	X							1
CYP19A1	15	cytochrome P450 family 19 subfamily A member 1					X			1
CYR61	1	cysteine rich angiogenic inducer 61	X							1
DAB2	5	DAB2, clathrin adaptor protein		X	X	X	X			4
DACH1	13	dachshund family transcription factor 1						X		1
DAXX	6	death domain associated protein	X		X	X		X		4
DCAF7	17	DDB1 and CUL4 associated factor 7					X			1
DCN	12	decorin	X							1
DDX5	17	DEAD-box helicase 5					X			1
DEDD	1	death effector domain containing					X			1
DEPTOR	8	DEP domain containing MTOR interacting protein					X			1
DIO3	14	iodothyronine deiodinase 3						X		1
DKK1	10	dickkopf WNT signaling pathway inhibitor 1	X							1
DLX1	2	distal-less homeobox 1					X	X		2
DOCK9	13	dedicator of cytokinesis 9				X	X			2
DPYSL2	8	dihydropyrimidinase like 2						X		1
DRAP1	11	DR1 associated protein 1				X	X	X		3
DROSHA	5	drosha ribonuclease III					X			1
DVL1	1	dishevelled segment polarity protein 1				X	X	X	X	4

DYNLRB1	20	dynein light chain roadblock-type 1		X	X	X				3
E2F4	16	E2F transcription factor 4				X	X	X		3
E2F5	8	E2F transcription factor 5				X	X	X		3
EBAG9	8	estrogen receptor binding site associated, antigen, 9						X		1
EDN1	6	endothelin 1	X							1
EGF	4	epidermal growth factor	X							1
EGR1	5	early growth response 1	X	X					X	3
EID2	19	EP300 interacting inhibitor of differentiation 2				X	X	X		3
EIF2A	3	eukaryotic translation initiation factor 2A		X	X					2
EIF2AK4	15	eukaryotic translation initiation factor 2 alpha kinase 4						X		1
EIF3I	1	eukaryotic translation initiation factor 3 subunit I	X							1
EIF4A3	17	eukaryotic translation initiation factor 4A3						X		1
EIF4ENIF1	22	eukaryotic translation initiation factor 4E nuclear import factor 1					X			1
ELAC2	17	elaC ribonuclease Z 2				X	X			2
ELANE	19	elastase, neutrophil expressed	X							1
ENG	9	endoglin	X	X	X		X	X		5
EOMES	3	eomesodermin	X							1
EP300	22	E1A binding protein p300		X		X	X	X	X	5
EPAS1	2	endothelial PAS domain protein 1						X		1
EPB41	1	erythrocyte membrane protein band 4.1					X			1
EPB41L5	2	erythrocyte membrane protein band 4.1 like 5				X	X			2
EPS15	1	epidermal growth factor receptor pathway substrate 15	X							1

ERBB2	17	erb-b2 receptor tyrosine kinase 2							X	1
ERC1	12	ELKS/RAB6-interacting/CAST family member 1		X	X					2
ERCC2	19	ERCC excision repair 2, TFIIH core complex helicase subunit		X	X					2
ERCC3	2	ERCC excision repair 3, TFIIH core complex helicase subunit		X	X					2
ESR1	6	estrogen receptor 1				X	X	X		3
ETV4	17	ETS variant 4				X				1
F10	13	coagulation factor X				X				1
F13A1	6	coagulation factor XIII A chain	X							1
F5	1	coagulation factor V	X							1
FAM83G	17	family with sequence similarity 83 member G				X	X			2
FASLG	1	Fas ligand	X							1
FBLN5	14	fibulin 5	X							1
FBN1	15	fibrillin 1	X							1
FCN1	9	ficolin 1	X							1
FGF2	4	fibroblast growth factor 2	X							1
FGF21	19	fibroblast growth factor 21	X							1
FHL2	2	four and a half LIM domains 2				X	X	X		3
FHL3	1	four and a half LIM domains 3				X	X	X		3
FKBP1A	20	FK506 binding protein 1A	X	X	X	X	X		X	6
FKBP2	11	FK506 binding protein 2				X				1
FLI1	11	Fli-1 proto-oncogene, ETS transcription factor			X					1
FMOD	1	fibromodulin	X							1
FN1	2	fibronectin 1	X							1
FNTA	8	farnesyltransferase, CAAX box, alpha	X	X						2
FOS	14	Fos proto-oncogene, AP-1 transcription factor subunit	X			X	X	X	X	5

FOXG1	14	forkhead box G1				X	X	X		3
FOXH1	8	forkhead box H1				X	X	X		3
FOXL2	3	forkhead box L2					X			1
FOXM1	12	forkhead box M1					X			1
FOXO1	13	forkhead box O1				X	X	X		3
FOXO3	6	forkhead box O3				X	X	X		3
FOXQ1	6	forkhead box Q1	X							1
FSHR	2	follicle stimulating hormone receptor	X							1
FST	5	follistatin	X							1
FSTL1	3	follistatin like 1	X							1
FURIN	15	furin, paired basic amino acid cleaving enzyme	X							1
GAS6	13	growth arrest specific 6	X							1
GAST	17	gastrin					X		X	2
GATA3	10	GATA binding protein 3	X				X	X		3
GATA4	8	GATA binding protein 4				X	X			2
GATA6	18	GATA binding protein 6				X				1
GDF11	12	growth differentiation factor 11		X						1
GDF9	5	growth differentiation factor 9		X		X				2
GJA1	6	gap junction protein alpha 1	X							1
GLI3	7	GLI family zinc finger 3						X		1
GNAQ	9	G protein subunit alpha q	X							1
GPC1	2	glypican 1		X	X	X				3
GRIP1	12	glutamate receptor interacting protein 1					X			1
GSC	14	goosecoid homeobox				X	X	X		3
GSK3B	3	glycogen synthase kinase 3 beta					X			1
GTF2H1	11	general transcription factor IIH subunit 1		X	X					2
GTF2H2	5	general transcription factor IIH subunit 2		X	X					2

GTF2H3	12	general transcription factor IIH subunit 3		X	X					2
GTF2I	7	general transcription factor IIi				X		X		2
HBZ	16	hemoglobin subunit zeta					X			1
HDAC1	1	histone deacetylase 1				X	X	X	X	4
HDAC2	6	histone deacetylase 2				X	X	X	X	4
HDAC3	5	histone deacetylase 3							X	1
HDAC4	2	histone deacetylase 4					X			1
HDAC5	17	histone deacetylase 5					X		X	2
HDAC9	7	histone deacetylase 9					X			1
HERC5	4	HECT and RLD domain containing E3 ubiquitin protein ligase 5						X		1
HGS	17	hepatocyte growth factor-regulated tyrosine kinase substrate				X	X	X		3
HIF1A	14	hypoxia inducible factor 1 alpha subunit					X	X		2
HIPK2	7	homeodomain interacting protein kinase 2				X	X			2
HIVEP1	6	human immunodeficiency virus type I enhancer binding protein 1		X						1
HMGA2	12	high mobility group AT-hook 2				X	X	X		3
HMMR	5	hyaluronan mediated motility receptor		X						1
HMOX1	22	heme oxygenase 1							X	1
HNF1A	12	HNF1 homeobox A						X		1
HNF4A	20	hepatocyte nuclear factor 4 alpha					X	X		2
HOXA13	7	homeobox A13				X				1
HOXA9	7	homeobox A9				X		X		2
HOXC11	12	homeobox C11					X			1
HOXC8	12	homeobox C8						X		1
HOXD13	2	homeobox D13				X				1
HRG	3	histidine rich glycoprotein	X							1

HRH1	3	histamine receptor H1				X	X			2
HSD17B1	17	hydroxysteroid 17-beta dehydrogenase 1	X							1
HSP90AA1	14	heat shock protein 90 alpha family class A member 1		X	X					2
HSPA4	5	heat shock protein family A (Hsp70) member 4		X		X	X			3
HSPA8	11	heat shock protein family A (Hsp70) member 8				X	X	X		3
HSPB1	7	heat shock protein family B (small) member 1	X							1
HSPB2-C11orf52	11	HSPB2-C11orf52 readthrough (NMD candidate)	X							1
HSPB3	5	heat shock protein family B (small) member 3	X							1
HSPG2	1	heparan sulfate proteoglycan 2	X							1
HYAL2	3	hyaluronoglucosaminidase 2	X							1
ICAM1	19	intercellular adhesion molecule 1	X							1
ID1	20	inhibitor of DNA binding 1, HLH protein	X					X		2
ID2	2	inhibitor of DNA binding 2	X							1
IFNA1	9	interferon alpha 1	X							1
IFNB1	9	interferon beta 1					X	X		2
IFNG	12	interferon gamma	X							1
IGF1	12	insulin like growth factor 1	X							1
IGF2	11	insulin like growth factor 2	X							1
IGFBP1	7	insulin like growth factor binding protein 1	X							1
IGFBP3	7	insulin like growth factor binding protein 3	X							1
IKBKB	8	inhibitor of nuclear factor kappa B kinase subunit beta		X	X					2
IL10	1	interleukin 10					X	X	X	3

IL13	5	interleukin 13	X			X				2
IL17A	6	interleukin 17A	X							1
IL1A	2	interleukin 1 alpha	X							1
IL1B	2	interleukin 1 beta			X				X	2
IL2	4	interleukin 2	X							1
IL25	14	interleukin 25				X				1
IL36RN	2	interleukin 36 receptor antagonist					X			1
IL37	2	interleukin 37					X			1
IL5	5	interleukin 5					X	X		2
IL6	7	interleukin 6	X							1
ING2	4	inhibitor of growth family member 2				X				1
INHBA	7	inhibin beta A subunit	X							1
INHBB	2	inhibin beta B subunit	X							1
INHBC	12	inhibin beta C subunit	X							1
INHBE	12	inhibin beta E subunit	X							1
INS	11	insulin	X						X	2
INSR	19	insulin receptor				X				1
IPO7	11	importin 7				X				1
IQGAP1	15	IQ motif containing GTPase activating protein 1			X					1
IRAK3	12	interleukin 1 receptor associated kinase 3	X							1
IRF3	19	interferon regulatory factor 3				X		X		2
IRF7	11	interferon regulatory factor 7				X	X	X		3
IRX3	16	iroquois homeobox 3					X			1
IRX6	16	iroquois homeobox 6					X			1
ITCH	20	itchy E3 ubiquitin protein ligase		X		X	X		X	4
ITGA5	12	integrin subunit alpha 5		X						1
ITGAE	17	integrin subunit alpha E	X							1
ITGAV	2	integrin subunit alpha V		X	X					2

ITGB5	3	integrin subunit beta 5					X	X		2
ITGB8	7	integrin subunit beta 8		X						1
JPH1	8	junctophilin 1					X			1
JPH3	16	junctophilin 3					X			1
JUN	1	Jun proto-oncogene, AP-1 transcription factor subunit	X			X	X	X	X	5
JUNB	19	JunB proto-oncogene, AP-1 transcription factor subunit				X	X	X		3
JUND	19	JunD proto-oncogene, AP-1 transcription factor subunit					X			1
KAT2B	3	lysine acetyltransferase 2B				X	X	X	X	4
KDM6B	17	lysine demethylase 6B				X	X	X		3
KLC1	14	kinesin light chain 1						X		1
KLF10	8	Kruppel like factor 10	X						X	2
KLF11	2	Kruppel like factor 11					X		X	2
KLF2	19	Kruppel like factor 2				X			X	2
KLF4	9	Kruppel like factor 4				X		X		2
KLF5	13	Kruppel like factor 5				X	X	X		3
KLF6	10	Kruppel like factor 6	X							1
KLHL9	9	kelch like family member 9					X			1
KNG1	3	kininogen 1	X							1
KPNB1	17	karyopherin subunit beta 1					X	X		2
KRT10	17	keratin 10					X			1
KRT2	12	keratin 2					X			1
LAMC1	1	laminin subunit gamma 1					X	X		2
LCK	1	LCK proto-oncogene, Src family tyrosine kinase				X	X			2
LEF1	4	lymphoid enhancer binding factor 1				X	X	X	X	4
LEFTY1	1	left-right determination factor 1					X			1
LEFTY2	1	left-right determination factor 2	X							1
LEMD3	12	LEM domain containing 3				X		X		2

LEP	7	leptin		X						1
LMNA	1	lamin A/C					X			1
LMO4	1	LIM domain only 4						X		1
LOX	5	lysyl oxidase					X			1
LRCH1	13	leucine rich repeats and calponin homology domain containing 1					X			1
LRG1	19	leucine rich alpha-2-glycoprotein 1		X	X					2
LRRC32	11	leucine rich repeat containing 32	X							1
LTBP1	2	latent transforming growth factor beta binding protein 1	X							1
LTBP4	19	latent transforming growth factor beta binding protein 4	X							1
LTC4S	5	leukotriene C4 synthase	X							1
LUM	12	lumican		X						1
MAD1L1	7	mitotic arrest deficient 1 like 1				X				1
MAD2L2	1	mitotic arrest deficient 2 like 2				X				1
MAFK	7	MAF bZIP transcription factor K					X			1
MAML1	5	mastermind like transcriptional coactivator 1					X			1
MAP2K1	15	mitogen-activated protein kinase kinase 1					X			1
MAP2K3	17	mitogen-activated protein kinase kinase 3							X	1
MAP2K6	17	mitogen-activated protein kinase kinase 6							X	1
MAP3K1	5	mitogen-activated protein kinase kinase kinase 1				X				1
MAP3K10	19	mitogen-activated protein kinase kinase kinase 10						X		1
MAP3K7	6	mitogen-activated protein kinase kinase kinase 7	X	X	X				X	4
MAPK1	22	mitogen-activated protein kinase 1	X			X		X		3

MAPK14	6	mitogen-activated protein kinase 14		X			X		X	3
MAPK3	16	mitogen-activated protein kinase 3	X			X	X	X		4
MAPK8	10	mitogen-activated protein kinase 8					X	X		2
MAX	14	MYC associated factor X				X	X	X		3
MCAM	11	melanoma cell adhesion molecule	X							1
MDM4	1	MDM4, p53 regulator					X	X		2
MECOM	3	MDS1 and EVI1 complex locus				X	X	X		3
MED15	22	mediator complex subunit 15				X	X	X		3
MED24	17	mediator complex subunit 24				X	X			2
MED6	14	mediator complex subunit 6				X	X			2
MEF2A	15	myocyte enhancer factor 2A				X	X			2
MEF2C	5	myocyte enhancer factor 2C				X	X	X		3
MEN1	11	menin 1				X	X	X		3
MIA	19	melanoma inhibitory activity	X							1
MITF	3	melanogenesis associated transcription factor					X			1
MKL1	22	megakaryoblastic leukemia (translocation) 1					X			1
MLTK	2	mitogen-activated protein kinase kinase kinase 20		X						1
MME	3	membrane metalloendopeptidase	X							1
MMP1	11	matrix metallopeptidase 1					X			1
MMP2	16	matrix metallopeptidase 2	X							1
MMP20	11	matrix metallopeptidase 20		X						1
MMP25	16	matrix metallopeptidase 25		X						1
MMP9	20	matrix metallopeptidase 9	X				X	X		3
MMRN1	4	multimerin 1	X							1
MMS19	10	MMS19 homolog, cytosolic iron-sulfur assembly component		X	X					2
MNAT1	14	MNAT1, CDK activating kinase assembly factor		X	X					2
MOXD1	6	monooxygenase DBH like 1							X	1

MSH2	2	mutS homolog 2	X							1
MST1R	3	macrophage stimulating 1 receptor						X		1
MTMR4	17	myotubularin related protein 4				X	X			2
MUC2	11	mucin 2, oligomeric mucus/gel-forming					X	X		2
MUC4	3	mucin 4, cell surface associated	X							1
MYC	8	MYC proto-oncogene, bHLH transcription factor	X			X	X	X	X	5
MYCN	2	MYCN proto-oncogene, bHLH transcription factor						X		1
MYO5B	18	myosin VB					X			1
MYOC	1	myocilin		X						1
MYOCD	17	myocardin				X	X	X		3
MYOD1	11	myogenic differentiation 1						X	X	2
NANOG	12	Nanog homeobox						X		1
NCAM1	11	neural cell adhesion molecule 1							X	1
NCBP1	9	nuclear cap binding protein subunit 1						X		1
NCOA1	2	nuclear receptor coactivator 1					X	X		2
NCOA3	20	nuclear receptor coactivator 3				X	X			2
NCOA6	20	nuclear receptor coactivator 6	X			X				2
NCOR1	17	nuclear receptor corepressor 1				X	X	X		3
NCOR2	12	nuclear receptor corepressor 2				X	X	X		3
NDRG2	14	NDRG family member 2					X			1
NEDD4	15	neural precursor cell expressed, developmentally down-regulated 4, E3 ubiquitin protein ligase				X	X		X	3
NEDD4L	18	neural precursor cell expressed, developmentally down-regulated 4-like, E3 ubiquitin protein ligase	X	X	X	X	X	X	X	7
NEDD8	14	neural precursor cell expressed, developmentally down-regulated 8			X					1

NEDD9	6	neural precursor cell expressed, developmentally down-regulated 9				X	X			2
NF1	17	neurofibromin 1				X	X	X		3
NFE2L2	2	nuclear factor, erythroid 2 like 2					X			1
NFIC	19	nuclear factor I C					X			1
NFKB1	4	nuclear factor kappa B subunit 1	X							1
NFYC	1	nuclear transcription factor Y subunit gamma				X	X			2
NGFR	17	nerve growth factor receptor						X		1
NKX2-1	14	NK2 homeobox 1					X			1
NKX2-5	5	NK2 homeobox 5				X	X	X		3
NKX3-2	4	NK3 homeobox 2						X		1
NLK	17	nemo like kinase						X		1
NODAL	10	nodal growth differentiation factor				X	X			2
NOS3	7	nitric oxide synthase 3	X			X				2
NOTCH1	9	notch 1		X			X			2
NOTCH3	19	notch 3				X	X	X		3
NOX4	11	NADPH oxidase 4	X							1
NR2C2	3	nuclear receptor subfamily 2 group C member 2	X	X	X					3
NR2F2	15	nuclear receptor subfamily 2 group F member 2						X		1
NR3C1	5	nuclear receptor subfamily 3 group C member 1					X	X		2
NR4A1	12	nuclear receptor subfamily 4 group A member 1							X	1
NR5A1	9	nuclear receptor subfamily 5 group A member 1						X		1
NRP1	10	neuropilin 1		X	X					2
NRP2	2	neuropilin 2			X					1
NUP153	6	nucleoporin 153				X	X	X		3
NUP214	9	nucleoporin 214				X	X	X		3

OCLN	5	occludin		X	X					2
ODC1	2	ornithine decarboxylase 1					X			1
OLIG1	21	oligodendrocyte transcription factor 1				X	X			2
OMD	9	osteomodulin	X							1
OPA1	3	OPA1, mitochondrial dynamin like GTPase				X	X			2
OTUB1	11	OTU deubiquitinase, ubiquitin aldehyde binding 1				X	X	X		3
PAEP	9	progestagen associated endometrial protein					X			1
PAK1	11	p21 (RAC1) activated kinase 1				X				1
PAK2	3	p21 (RAC1) activated kinase 2				X				1
PAK4	19	p21 (RAC1) activated kinase 4				X				1
PARD3	10	par-3 family cell polarity regulator	X	X	X	X	X	X	X	7
PARD3B	2	par-3 family cell polarity regulator beta				X	X	X	X	4
PARD6A	16	par-6 family cell polarity regulator alpha	X	X	X					3
PARP1	1	poly(ADP-ribose) polymerase 1				X	X	X		3
PAX6	11	paired box 6	X				X	X		3
PBX1	1	PBX homeobox 1						X		1
PCNA	20	proliferating cell nuclear antigen				X				1
PDGFB	22	platelet derived growth factor subunit B	X							1
PDGFRA	4	platelet derived growth factor receptor alpha	X							1
PDPK1	16	3-phosphoinositide dependent protein kinase 1		X	X				X	3
PEG10	7	paternally expressed 10		X	X					2
PEX6	6	peroxisomal biogenesis factor 6					X			1
PF4	4	platelet factor 4	X							1

PGR	11	progesterone receptor	X							1
PHKG2	16	phosphorylase kinase catalytic subunit gamma 2		X	X					2
PIAS1	15	protein inhibitor of activated STAT 1						X		1
PIAS2	18	protein inhibitor of activated STAT 2					X	X		2
PIAS4	19	protein inhibitor of activated STAT 4				X	X	X	X	4
PIK3CA	3	phosphatidylinositol-4,5-bisphosphate 3-kinase catalytic subunit alpha				X	X			2
PIK3R1	5	phosphoinositide-3-kinase regulatory subunit 1		X	X					2
PIK3R2	19	phosphoinositide-3-kinase regulatory subunit 2		X	X					2
PIN1	19	peptidylprolyl cis/trans isomerase, NIMA-interacting 1				X	X			2
PITX1	5	paired like homeodomain 1					X			1
PITX2	4	paired like homeodomain 2					X			1
PKNOX1	21	PBX/knotted 1 homeobox 1				X				1
PLG	6	plasminogen	X							1
PLOD1	1	procollagen-lysine,2-oxoglutarate 5-dioxygenase 1	X							1
PMEPA1	20	prostate transmembrane protein, androgen induced 1				X	X			2
PML	15	promyelocytic leukemia		X	X	X	X		X	5
POSTN	13	periostin	X							1
POU5F1	6	POU class 5 homeobox 1						X		1
PPARA	22	peroxisome proliferator activated receptor alpha						X		1
PPARD	6	peroxisome proliferator activated receptor delta						X		1

PPARG	3	peroxisome proliferator activated receptor gamma	X			X				2
PPBP	4	pro-platelet basic protein	X							1
PPM1A	14	protein phosphatase, Mg2+/Mn2+ dependent 1A				X	X	X		3
PPP1CA	11	protein phosphatase 1 catalytic subunit alpha	X	X	X				X	4
PPP1CB	2	protein phosphatase 1 catalytic subunit beta	X	X	X				X	4
PPP1CC	12	protein phosphatase 1 catalytic subunit gamma	X	X	X				X	4
PPP1R15A	19	protein phosphatase 1 regulatory subunit 15A	X	X	X				X	4
PPP2CA	5	protein phosphatase 2 catalytic subunit alpha			X					1
PPP2CB	8	protein phosphatase 2 catalytic subunit beta			X					1
PPP2R1A	19	protein phosphatase 2 scaffold subunit Aalpha			X					1
PPP2R1B	11	protein phosphatase 2 scaffold subunit Abeta			X					1
PPP2R2A	8	protein phosphatase 2 regulatory subunit Balpha		X	X					2
PPP2R5D	6	protein phosphatase 2 regulatory subunit B'delta	X	X	X					3
PRDM14	8	PR/SET domain 14						X		1
PRDM16	1	PR/SET domain 16				X	X			2
PRG4	1	proteoglycan 4	X							1
PRKAA1	5	protein kinase AMP-activated catalytic subunit alpha 1		X	X					2
PRKAB1	12	protein kinase AMP-activated non-catalytic subunit beta 1			X					1

PRKACA	19	protein kinase cAMP-activated catalytic subunit alpha		X	X				2
PRKACB	1	protein kinase cAMP-activated catalytic subunit beta		X	X				2
PRKAG1	12	protein kinase AMP-activated non-catalytic subunit gamma 1		X	X				2
PRKAG2	7	protein kinase AMP-activated non-catalytic subunit gamma 2		X	X				2
PRKAG3	2	protein kinase AMP-activated non-catalytic subunit gamma 3		X	X				2
PRKAR1A	17	protein kinase cAMP-dependent type I regulatory subunit alpha		X	X				2
PRKAR1B	7	protein kinase cAMP-dependent type I regulatory subunit beta		X	X				2
PRKAR2A	3	protein kinase cAMP-dependent type II regulatory subunit alpha		X	X				2
PRKCZ	1	protein kinase C zeta	X	X	X				3
PRKG1	10	protein kinase, cGMP-dependent, type I						X	1
PRL	6	prolactin	X						1
PROC	2	protein C, inactivator of coagulation factors Va and VIIIa					X	X	2
PROM1	4	prominin 1	X						1
PROS1	3	protein S	X						1
PRTN3	19	proteinase 3					X		1
PSEN1	14	presenilin 1		X					1
PSPC1	13	paraspeckle component 1					X		1
PTEN	10	phosphatase and tensin homolog	X				X	X	3
PTGS2	1	prostaglandin-endoperoxide synthase 2	X						1
PTH	11	parathyroid hormone					X		1
PTHLH	12	parathyroid hormone like hormone	X						1
PTK2	8	protein tyrosine kinase 2	X						1

PTPN14	1	protein tyrosine phosphatase, non-receptor type 14	X							1
PTPRK	6	protein tyrosine phosphatase, receptor type K				X				1
PYCARD	16	PYD and CARD domain containing		X	X					2
PYDC1	16	pyrin domain containing 1		X	X					2
PYGL	14	glycogen phosphorylase L					X			1
PZP	12	PZP, alpha-2-macroglobulin like	X							1
RANBP3	19	RAN binding protein 3				X	X			2
RANBP9	6	RAN binding protein 9		X						1
RAPGEF3	12	Rap guanine nucleotide exchange factor 3		X	X					2
RAPGEF4	2	Rap guanine nucleotide exchange factor 4		X	X					2
RARG	12	retinoic acid receptor gamma					X			1
RB1CC1	8	RB1 inducible coiled-coil 1							X	1
RBBP4	1	RB binding protein 4, chromatin remodeling factor				X	X	X		3
RBL1	20	RB transcriptional corepressor like 1				X	X	X		3
RBPMS	8	RNA binding protein, mRNA processing factor						X		1
RBX1	22	ring-box 1				X	X			2
RELA	11	RELA proto-oncogene, NF-kB subunit	X						X	2
RGCC	13	regulator of cell cycle				X	X			2
RGMA	15	repulsive guidance molecule BMP co-receptor a							X	1
RGMB	5	repulsive guidance molecule BMP co-receptor b							X	1
RHOA	3	ras homolog family member A	X	X	X					3
RNF11	1	ring finger protein 11						X		1

RNF111	15	ring finger protein 111				X	X		X	3
RNF165	18	ring finger protein 165							X	1
RORC	1	RAR related orphan receptor C	X							1
RPS27A	2	ribosomal protein S27a	X	X	X	X	X	X	X	7
RPS6KB1	17	ribosomal protein S6 kinase B1	X	X	X					3
RSPH3	6	radial spoke head 3 homolog					X			1
RUNX1	21	runt related transcription factor 1				X	X	X		3
RUNX2	6	runt related transcription factor 2				X	X	X		3
RUNX3	1	runt related transcription factor 3				X	X	X		3
S100A4	1	S100 calcium binding protein A4				X	X			2
SALL4	20	spalt like transcription factor 4						X		1
SAP18	13	Sin3A associated protein 18				X	X	X		3
SAP30	4	Sin3A associated protein 30				X	X	X		3
SAR1A	10	secretion associated Ras related GTPase 1A	X	X	X	X			X	5
SDC1	2	syndecan 1	X							1
SDC2	8	syndecan 2	X							1
SERPINA1	14	serpin family A member 1	X							1
SERPINE1	7	serpin family E member 1	X	X		X	X	X	X	6
SERPINF2	17	serpin family F member 2	X							1
SERPING1	11	serpin family G member 1	X							1
SETDB1	1	SET domain bifurcated 1					X			1
SF3A1	22	splicing factor 3a subunit 1					X			1
SF3B2	11	splicing factor 3b subunit 2					X			1
SFTPB	2	surfactant protein B					X			1
SH2D2A	1	SH2 domain containing 2A				X	X			2
SHC1	1	SHC adaptor protein 1		X	X					2
SIK3	11	SIK family kinase 3				X	X			2
SIN3A	15	SIN3 transcription regulator family member A				X	X	X		3

SIN3B	19	SIN3 transcription regulator family member B				X	X	X		3
SIRT1	10	sirtuin 1					X		X	2
SKI	1	SKI proto-oncogene		X		X	X	X		4
SKIL	3	SKI like proto-oncogene		X		X	X	X	X	5
SKP1	5	S-phase kinase associated protein 1				X				1
SKP2	5	S-phase kinase associated protein 2						X	X	2
SLC25A12	2	solute carrier family 25 member 12					X			1
SLC26A3	7	solute carrier family 26 member 3	X							1
SLC29A1	6	solute carrier family 29 member 1 (Augustine blood group)	X							1
SLC2A1	1	solute carrier family 2 member 1							X	1
SLC5A2	16	solute carrier family 5 member 2	X							1
SLC5A4	22	solute carrier family 5 member 4	X							1
SLC6A1	3	solute carrier family 6 member 1						X		1
SLPI	20	secretory leukocyte peptidase inhibitor	X							1
SMAD1	4	SMAD family member 1	X		X	X	X	X	X	6
SMAD2	18	SMAD family member 2	X	X	X		X	X	X	7
SMAD3	15	SMAD family member 3	X	X	X	X		X	X	7
SMAD4	18	SMAD family member 4	X		X	X	X		X	6
SMAD5	5	SMAD family member 5	X		X			X	X	4
SMAD6	15	SMAD family member 6	X		X	X		X	X	5
SMAD7	18	SMAD family member 7	X	X	X	X	X	X		7
SMAD9	13	SMAD family member 9	X		X	X		X	X	5
SMARCA4	19	SWI/SNF related, matrix associated, actin dependent regulator of chromatin, subfamily a, member 4				X	X			2
SMARCC1	3	SWI/SNF related, matrix associated, actin dependent				X	X			2

		regulator of chromatin subfamily c member 1								
SMARCC2	12	SWI/SNF related, matrix associated, actin dependent regulator of chromatin subfamily c member 2				X	X			2
SMURF1	7	SMAD specific E3 ubiquitin protein ligase 1	X	X	X	X	X		X	6
SMURF2	17	SMAD specific E3 ubiquitin protein ligase 2				X	X		X	3
SNAI1	20	snail family transcriptional repressor 1	X				X	X		3
SNAI2	8	snail family transcriptional repressor 2			X					1
SNIP1	1	Smad nuclear interacting protein 1	X			X	X	X		4
SNW1	14	SNW domain containing 1				X	X	X		3
SNX6	14	sorting nexin 6		X	X					2
SOST	17	sclerostin	X							1
SOX2	3	SRY-box 2	X					X		2
SOX9	17	SRY-box 9				X	X			2
SP1	12	Sp1 transcription factor				X	X	X	X	4
SP3	2	Sp3 transcription factor					X	X		2
SP7	12	Sp7 transcription factor						X		1
SPARC	5	secreted protein acidic and cysteine rich	X							1
SPHK1	17	sphingosine kinase 1				X				1
SPP1	4	secreted phosphoprotein 1	X							1
SPRY2	13	sprouty RTK signaling antagonist 2	X							1
SPTBN1	2	spectrin beta, non-erythrocytic 1		X	X	X	X	X		5
SRC	20	SRC proto-oncogene, non-receptor tyrosine kinase			X		X			2
SRCIN1	17	SRC kinase signaling inhibitor 1						X		1

SREBF2	22	sterol regulatory element binding transcription factor 2					X			1
SRF	6	serum response factor				X			X	2
SRGN	10	serglycin	X							1
SS18L1	20	SS18L1, nBAF chromatin remodeling complex subunit					X			1
STAMBP	2	STAM binding protein							X	1
STAT1	2	signal transducer and activator of transcription 1				X	X		X	3
STAT3	17	signal transducer and activator of transcription 3							X	1
STAT5A	17	signal transducer and activator of transcription 5A					X			1
STAT5B	17	signal transducer and activator of transcription 5B					X			1
STK11	19	serine/threonine kinase 11						X		1
STK11IP	2	serine/threonine kinase 11 interacting protein						X		1
STRAP	12	serine/threonine kinase receptor associated protein	X	X	X	X	X		X	6
STUB1	16	STIP1 homology and U-box containing protein 1				X	X	X		3
SUMO1	2	small ubiquitin-like modifier 1					X	X		2
TAB1	22	TGF-beta activated kinase 1 (MAP3K7) binding protein 1		X	X				X	3
TAB2	6	TGF-beta activated kinase 1/MAP3K7 binding protein 2		X	X				X	3
TAC1	7	tachykinin precursor 1	X							1
TBX6	16	T-box 6						X		1
TCF12	15	transcription factor 12				X				1
TCF4	18	transcription factor 4							X	1
TCF7	5	transcription factor 7						X		1
TCF7L1	2	transcription factor 7 like 1						X		1

TCF7L2	10	transcription factor 7 like 2						X		1
TCTEX1D4	1	Tctex1 domain containing 4			X					1
TDGF1	3	teratocarcinoma-derived growth factor 1							X	1
TERT	5	telomerase reverse transcriptase			X		X			2
TFDP1	13	transcription factor Dp-1				X	X	X		3
TFDP2	3	transcription factor Dp-2				X	X	X		3
TFEB	6	transcription factor EB							X	1
TGFB1	19	transforming growth factor beta 1		X	X	X	X	X	X	7
TGFB1I1	16	transforming growth factor beta 1 induced transcript 1					X	X	X	3
TGFB2	1	transforming growth factor beta 2	X	X	X	X	X	X		6
TGFB3	14	transforming growth factor beta 3	X	X	X	X	X	X		6
TGFBR1	9	transforming growth factor beta receptor 1	X		X	X	X		X	6
TGFBR2	3	transforming growth factor beta receptor 2	X	X		X	X	X	X	7
TGFBR3	1	transforming growth factor beta receptor 3	X	X	X					3
TGFBRAP1	2	transforming growth factor beta receptor associated protein 1	X	X	X	X		X		5
TGIF1	18	TGFB induced factor homeobox 1				X	X	X		3
TGIF2	20	TGFB induced factor homeobox 2				X	X	X		3
THBS1	15	thrombospondin 1	X					X		2
THOC5	22	THO complex 5						X		1
TIMP3	22	TIMP metallopeptidase inhibitor 3	X							1
TJP1	15	tight junction protein 1	X							1
TLE1	9	transducin like enhancer of split 1						X		1
TLX2	2	T cell leukemia homeobox 2						X		1
TNFRSF11B	8	TNF receptor superfamily member 11b	X							1
TNFSF13B	13	TNF superfamily member 13b						X		1

TNK1	17	tyrosine kinase non receptor 1						X		1
TOB1	17	transducer of ERBB2, 1					X	X		2
TOLLIP	11	toll interacting protein		X					X	2
TP53	17	tumor protein p53	X			X	X	X	X	5
TP63	3	tumor protein p63					X			1
TPM1	15	tropomyosin 1		X						1
TPM3	1	tropomyosin 3					X			1
TRAF4	17	TNF receptor associated factor 4		X	X					2
TRAF6	11	TNF receptor associated factor 6		X	X					2
TRAP1	16	TNF receptor associated protein 1		X						1
TRIB3	20	tribbles pseudokinase 3					X			1
TRIM33	1	tripartite motif containing 33				X	X	X		3
TRIM62	1	tripartite motif containing 62					X			1
TSC2	16	TSC complex subunit 2				X	X			2
TSC22D1	13	TSC22 domain family member 1		X	X				X	3
TTK	6	TTK protein kinase				X				1
TWIST1	7	twist family bHLH transcription factor 1						X		1
TXN	9	thioredoxin							X	1
U2AF2	19	U2 small nuclear RNA auxiliary factor 2				X				1
UBA52	19	ubiquitin A-52 residue ribosomal protein fusion product 1	X	X	X	X	X	X	X	7
UBB	17	ubiquitin B	X	X	X	X	X	X	X	7
UBC	12	ubiquitin C	X	X	X	X	X	X	X	7
UBE2D1	10	ubiquitin conjugating enzyme E2 D1				X	X	X		3
UBE2M	19	ubiquitin conjugating enzyme E2 M			X					1
UBE2O	17	ubiquitin conjugating enzyme E2 O							X	1
UCHL5	1	ubiquitin C-terminal hydrolase L5	X	X	X	X	X		X	6
UCN	2	urocortin	X							1

UCP2	11	uncoupling protein 2						X		1
USP15	12	ubiquitin specific peptidase 15	X	X	X	X	X	X	X	7
USP4	3	ubiquitin specific peptidase 4		X						1
VASN	16	vasorin	X							1
VDR	12	vitamin D receptor	X				X	X		3
VEGFA	6	vascular endothelial growth factor A	X					X		2
VEGFB	11	vascular endothelial growth factor B	X							1
VEGFC	4	vascular endothelial growth factor C	X							1
VIM	10	vimentin	X				X			2
VWF	12	von Willebrand factor	X							1
WISP2	20	WNT1 inducible signaling pathway protein 2	X							1
WNK1	12	WNK lysine deficient protein kinase 1				X				1
WNK4	17	WNK lysine deficient protein kinase 4				X				1
WNT1	12	Wnt family member 1						X		1
WT1	11	Wilms tumor 1	X							1
WWOX	16	WW domain containing oxidoreductase					X	X		2
WWP1	8	WW domain containing E3 ubiquitin protein ligase 1		X	X	X	X	X	X	6
WWP2	16	WW domain containing E3 ubiquitin protein ligase 2				X			X	2
WWTR1	3	WW domain containing transcription regulator 1				X	X	X	X	4
XPO1	2	exportin 1						X	X	2
XPO4	13	exportin 4					X			1
YAP1	11	Yes associated protein 1		X	X			X	X	4

YBX1	1	Y-box binding protein 1	X				X		X	3
YTHDF2	1	YTH N6-methyladenosine RNA binding protein 2					X			1
YWHAE	17	tyrosine 3-monooxygenase/tryptophan 5-monooxygenase activation protein epsilon	X							1
YY1	14	YY1 transcription factor	X			X	X	X	X	5
ZBTB17	1	zinc finger and BTB domain containing 17				X	X	X		3
ZBTB3	11	zinc finger and BTB domain containing 3					X			1
ZDHHC4	7	zinc finger DHHC-type containing 4					X			1
ZEB1	10	zinc finger E-box binding homeobox 1				X	X	X	X	4
ZEB2	2	zinc finger E-box binding homeobox 2				X	X			2
ZFYVE16	5	zinc finger FYVE-type containing 16		X	X	X		X		4
ZFYVE9	1	zinc finger FYVE-type containing 9		X	X	X	X		X	5
ZMIZ1	10	zinc finger MIZ-type containing 1					X	X		2
ZMYM2	13	zinc finger MYM-type containing 2					X			1
ZNF423	16	zinc finger protein 423						X		1
ZNF521	18	zinc finger protein 521						X		1
ZNF8	19	zinc finger protein 8				X	X	X		3
ZSCAN10	16	zinc finger and SCAN domain containing 10						X		1
Total of targeted genes per core genes			223	132	122	221	305	229	123	

([a]) We used The Human Gene Connectome to search for targeted genes connected to each of the 7 main core genes at a degree of connectivity of 1. We selected only autosomal genes. We ended up with a total of 707 genes connected to the TGFB pathway.
([b]) Number of main core genes connected to the target gene.

Supplementary table 2. Results of the rare variant association study between liver fibrosis and 116 autosomal genes with more than 5 carriers of candidate coding variants (CCVs).

Gene name	Total number of CCVs	Number of carriers			*P*-value(£)
		Severe liver fibrosis (N=48)	No liver fibrosis (N=40)	Total (N=88)	
ENG	3	7	0	7	0.0003767402
TCF7L1	3	10	1	11	0.004880177
ICAM1	3	2	8	10	0.01665771
AGL	5	8	1	9	0.0190327
TGFB1	1	2	8	10	0.02157767
ATM	9	4	10	14	0.03145805
CYP19A1	3	7	1	8	0.03300056
KDM6B	8	10	17	27	0.03541138
PRG4	5	3	8	11	0.05336971
TOLLIP	2	8	2	10	0.05526237
TJP1	2	6	1	7	0.06071706
EPS15	4	3	7	10	0.1253163
OPA1	9	11	4	15	0.1446664
FOXM1	3	5	1	6	0.1457352
ACTN1	3	2	5	7	0.1503467
NCAM1	6	20	11	31	0.1515251
PPP1CA	1	6	2	8	0.1600318
NCBP1	3	6	2	8	0.1683185
MUC2	13	15	8	23	0.1789459
RUNX2	4	11	15	26	0.1810995
BMPR2	2	6	2	8	0.1868056
NRP2	4	4	7	11	0.1971068
GLI3	11	8	11	19	0.20023
MYOCD	4	6	2	8	0.2021462
NOTCH3	12	11	5	16	0.2196094
EPB41L5	3	5	2	7	0.2342188
LAMC1	10	9	4	13	0.2475057
KRT10	1	20	22	42	0.2533108
BRCA2	12	7	9	16	0.2638085
ANK1	9	7	10	17	0.2642263
ARID1B	7	16	9	25	0.2651101
NOTCH1	14	8	11	19	0.265763
PTK2	4	3	5	8	0.2812634
CCL17	1	5	2	7	0.2824949
HNF1A	3	3	5	8	0.2932677
CD44	2	2	4	6	0.2994858

TLE1	2	5	2	7	0.3101249
CDC25A	2	2	4	6	0.3382606
SREBF2	3	5	2	7	0.3448401
SERPING1	2	3	5	8	0.3716798
YY1	2	6	3	9	0.3911472
SLC25A12	1	6	3	9	0.401652
PPARA	1	10	5	15	0.402628
CREBBP	7	7	4	11	0.4030689
CDH2	3	9	5	14	0.41599
MMRN1	7	5	6	11	0.421695
MCAM	5	7	4	11	0.425149
MYC	3	5	2	7	0.4254118
LTBP1	13	14	9	23	0.4796528
TSC22D1	2	4	2	6	0.4844154
MST1R	3	8	4	12	0.4915808
CDKN2A	1	4	2	6	0.499084
TERT	4	6	7	13	0.5004928
PRKAG3	3	5	6	11	0.5070674
ETV4	5	3	4	7	0.5458456
PYGL	4	4	5	9	0.5548177
HMMR	4	4	4	8	0.5624998
SS18L1	4	3	4	7	0.5700073
C6orf132	9	17	16	33	0.5725212
HIVEP1	8	9	6	15	0.5869608
FURIN	5	3	4	7	0.5873681
RSPH3	5	6	3	9	0.5920537
TSC2	9	8	5	13	0.6026516
HSPA4	4	5	5	10	0.6112336
PLG	4	5	2	7	0.6262203
MAML1	6	6	4	10	0.629702
FOS	2	5	5	10	0.6301627
CGN	12	11	11	22	0.6306096
ARNT	2	3	3	6	0.6329987
PTPN14	8	10	7	17	0.6351806
TRAP1	11	8	8	16	0.6424565
MYO5B	9	6	6	12	0.6464961
MMP9	4	3	3	6	0.6881425
DOCK9	4	8	5	13	0.7019432
NUP153	8	6	4	10	0.7048017
COL2A1	2	7	5	12	0.7115241
LTBP4	8	8	6	14	0.7225749
ADAMTS3	7	5	3	8	0.7315069
PARD6A	1	3	3	6	0.7393433

PARD3B	6	5	3	8	0.7411917
PRKG1	1	7	5	12	0.7475818
SLC26A3	4	3	3	6	0.750003
BTRC	1	3	3	6	0.7604756
F13A1	1	4	3	7	0.7678977
POSTN	7	5	5	10	0.7832856
BMP6	5	3	3	6	0.7906692
ITGB5	8	5	5	10	0.7940216
SERPINA1	3	4	3	7	0.7958404
IRX3	5	3	3	6	0.8021104
ZFYVE9	6	4	3	7	0.8038968
MUC4	5	3	3	6	0.8056764
NCOA3	5	14	13	27	0.8065463
HSPG2	38	25	21	46	0.8120555
TGIF1	4	19	14	33	0.8184385
SLC5A4	7	3	3	6	0.822418
SMAD3	1	7	6	13	0.8264372
PPP2R1B	5	8	6	14	0.8328207
NUP214	5	3	3	6	0.8363385
WISP2	3	5	4	9	0.8488772
NEDD4	5	5	5	10	0.8531652
SMARCA4	5	3	3	6	0.8570248
NCOA1	6	5	5	10	0.8615588
ID1	3	4	3	7	0.8664492
VWF	16	8	6	14	0.8716261
PPP1R15A	4	4	3	7	0.879495
IRAK3	5	8	6	14	0.8921585
F5	3	6	5	11	0.9004119
HNF4A	2	4	3	7	0.9091592
JPH3	6	3	3	6	0.9185293
BCL6	3	5	4	9	0.9199447
BRCA1	5	12	10	22	0.9545136
DEPTOR	3	5	4	9	0.9617026
SETDB1	3	7	5	12	0.9643937
MMS19	6	9	8	17	0.9714794
WNK1	6	4	3	7	0.9762689
MOXD1	3	5	4	9	0.9895341

(£) P-values were obtained using a dominant genetic mode of inheritance with adjustment on the 3 first principal component to take into account population structure.

Supplementary Table 3. Distribution of carriers and characteristics of *ENG* candidate coding variants (CCVs) in patients of the discovery cohort (N=88) and the French replication cohort (N=161).

ENG CCVs	rs id	ref	alt	Discovery cohort				French replication cohort				GnomAD (European)		CADD score
				No fibrosis n=40		Severe fibrosis, n=48		No fibrosis, n=102		Severe fibrosis, n=59				
				carriers (%)	MAF[a]	carriers (%)	MAF[a]	carriers (%)	MAF[a]	carriers (%)	MAF[a]	%carriers[b]	MAF	v1.3
p.Thr5Met	rs35400405	G	A	0 (0%)	0	4 (8.3%)	0.042	4 (3.9%)	0.020	6 (10.2%)	0.051	4.1%	0.021	3.86
p.Pro131Leu	rs139398993	G	A	0 (0%)	0	1 (2.1%)	0.010	-		-		0.3%	0.001	6.64
p.Gly191Asp	rs41322046	C	T	0 (0%)	0	2 (4.2%)	0.021	3 (2.94%)	0.015	1 (1.7%)	0.008	3.1%	0.016	23.9
p.Gly545Ser	rs142896669	C	T	-	-			1 (0.98%)	0.005	0 (0%)	0	0.1%	0.0005	29.9
p.Arg571His	rs138799379	C	T	-	-			1 (0.98%)	0.005	0 (0%)	0	0.02%	0.0001	20.7
p.Ser615Leu	rs148002300	G	A	-	-			1 (0.98%)	0.005	0 (0%)	0	0.05%	0.002	22.8

[a] Minor allele frequency. As all carriers in the discovery cohort and in the French replication cohort were heterozygous the MAF is half of the proportion of carriers.
[b] In the GnomAD public database including > 50,000 Europeans, 26 homozygous were reported for p.Thr5Met, 1 for p.Pro131Leu, 8 for p.Gly191Asp and none for the other *ENG* CCVs.

References

Belkadi, A., Pedergnana, V., Cobat, A., Itan, Y., Vincent, Q.B., Abhyankar, A., et al. (2016). Whole-exome sequencing to analyze population structure, parental inbreeding, and familial linkage. *Proc Natl Acad Sci U S A* 113(24), 6713-6718. doi: 10.1073/pnas.1606460113.
